# Carbon dioxide laser ablation as an effective method for treating nodular areas in Port wine stains: a series of two cases

**DOI:** 10.1007/s10103-025-04591-9

**Published:** 2025-08-15

**Authors:** Susanne Dr. med. Bauer, Wolfgang Prof. Dr. Bäumler, Bernadett Dr. med. Kurz, Mark Prof. Dr. Berneburg, Julian Dr. med. Kögel

**Affiliations:** University Hospital Regensburg, Regensburg, Germany

**Keywords:** PWS– nodular, Capillary malformation– dermatologic surgery, Carbon dioxide laser ablation

## Abstract

Port wine stain (PWS) is a vascular, benign congenital malformation, which presents at birth and persists for life. PWS usually appears as flat red macule but tend to darken progressively to purple with soft tissue hypertrophy and may often develop disfiguring vascular nodules leading to a reduced quality of life for patients. Therapeutic interventions include the use of different laser systems such as pulsed dye lasers. To report the removal of PWS nodules with CO_2_ laser. Two PWS patients with nodules were included in the study. A CO_2_ laser (Unilas Touch, Limmer) was used to vaporize the lesion under local anesthesia. Laser parameters were selected to minimize the risk of potential scarring. The follow up of the patients showed a significant improvement in the skin condition and the nodular areas of the PWS became flatter without notable side effects. The CO_2_ laser treatment is a safe and effective treatment modality to remove nodules in PWS leading to an improved appearance of facial PWS.

## Introduction

Port wine stain (PWS) is a benign vascular malformation primarily caused by a malformation in the capillaries and post-capillary venules. PWS is one of the most common congenital vascular anomalies, which presents at birth and persists for life and its prevalence is estimated at three to five children per 1000 births equivalent to about 26 million people worldwide with PWS birthmarks [1, 2].

The birthmark is mostly located in the head and neck region [3]. Especially in this region there is an increased association with syndromal diseases. Patients with manifestations of PWS in the area supplied by the trigeminal nerve (especially V1) have a significantly increased risk of ocular and/or central nervous system involvement [4, 5]. The symptom complex consisting of the presence of a PWS in the area of the trigeminal nerve supply, leptomeningeal angiomatosis and choroidal angiomatosis is also known as encephalotrigeminal angiomatosis oder Sturge-Weber syndrome (SWS) [6]. The incidence is approximately 3 in 100,000 patients per year. Complications can lead to the development of secondary glaucoma and an increased risk of central nervous system events such as epileptic seizures, hemiparesis, or stroke [6–8]. Due to the high association of facial PWS, particularly when the trigeminal nerve is affected, with SWS a neuro-ocular involvement should be excluded before starting any laser treatment. This requires an interdisciplinary cooperation with neurologist (contrast-enhanced MRI) and ophthalmologist (screening for glaucoma) [7, 9].

At the beginning, PWS appears as flat red macule but tend to darken following puberty [10]. In a certain proportion of patients, nodules in PWS may develop at an average age of 22 years (14–53 years). This leads to an increased disfigurement and to an increased reduction in quality of life [11]. According to older reports up to 60% o PWS located in the face will undergo a nodular transformation with increasing age [10]. Newer studies have estimated that nodules may appear in about 44% of ntreated adult patients and about 10% in mixed patient population of treated and untreated patients [12]. These variations are probably due to early laser intervention, which can delay the onset of PWS nodularity [13].

Histologically the initial erythema in patients with port wine stain correlates with a greater number of dilated vessel, which are mostly located in the superficial and middle dermal layer. Differences in histological structure can be observed in different types of port wine stain [2]. In hypertrophic and nodular PWS the diameter and depth of vessels was significantly greater compared to red or purple PWS. Whereas collagen and elastic fibres in red and purple PWS were packed as in normal skin (dense and uniform arrangement) hypertrophic and nodular PWS showed loose and thinner collagen fibres around vessels as well as a greater number of disorderly arranged elastic fibres. The latter showed a multi-layered packing within the vessels [2].

Regarding the pathophysiology, studies reported that the majority of patients with PWS as well as SWS show activating GNAQ mutation leading to vascular and soft tissue proliferation [14]. Ultrastructural investigations of hypertrophic and nodular PWS showed that hyperactive and proliferative endothelial cells (Ecs), pericytes and fibroblasts are present [15]. Other studies suggest that both PKCa and PI3K signaling pathways contribute to the development of hypertrophy and nodularity in adult PWS [13].

The gold standard of PWS treatment is the use of pulsed dye lasers [16]. For treatment-resistant cases, pulsed alexandrite lasers (755 nm) or high-energy flash lamps (555 nm) could be used. Also Nd: YAG lasers (1064 nm) can be recommended for the treatment of PWS [17]. Regarding the reasonably frequent occurrence of nodules in PWS, a satisfactory and safe therapy for nodule removal is important. While surgical approaches are outdated, CO_2_ laser ablation could be an alternative. We present two cases with nodular PWS treated with CO_2_-laser ablation.

## Case Presentation

### Case 1

**Fig. 1:**
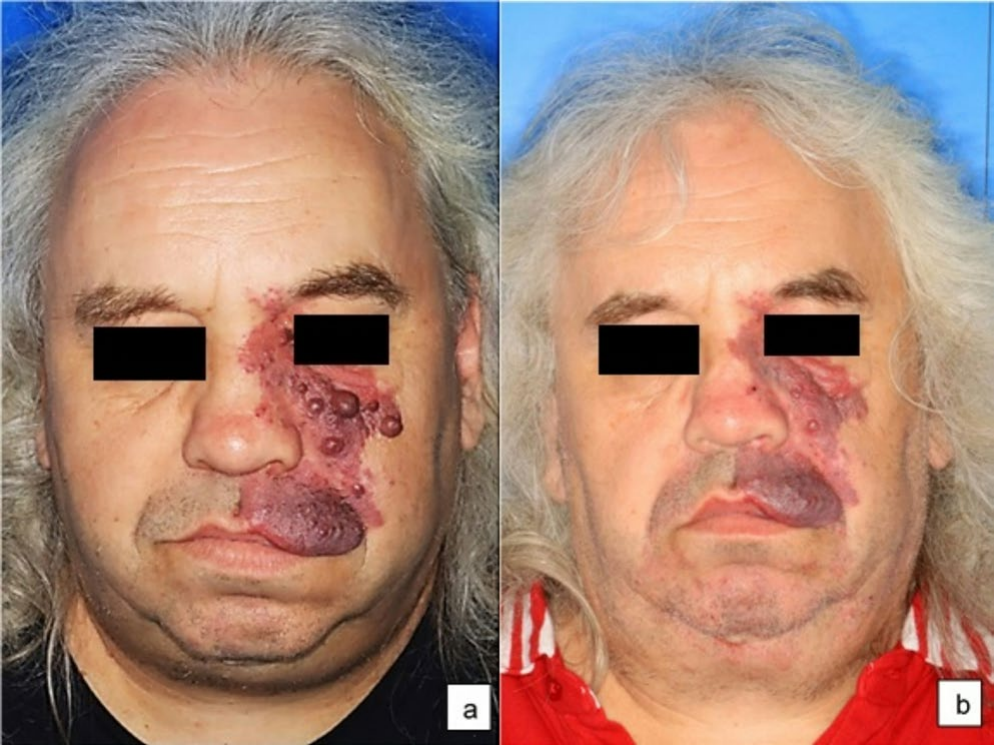
Case 1: A 58-year-old man with congenital PWS on the left side of his face. **a** Before treatment. **b** After treatment

In 2023, a patient presented with a PWS on the left side of the face (Fig. 1a). There were no indications for the presence of a SWS. Since November 1993, the patient had undergone multiple treatments using different pulsed dye lasers at wavelengths ranging from 585 nm to 600 nm [18]. However, these treatments did not result in complete remission of PWS. The patient also reported a reduction in his quality of life, particularly due to the PWS nodules that had appeared over time. Initially, an attempt was made to treat the nodules with a pulsed Nd: Yag laser at 1064 nm using a radiant exposure of 160 J/cm², a pulse duration of 90 ms, and a spot size of 3 mm. However, this did not substantially reduce the size of the nodules.

The patient was offered a treatment using CO2 laser ablation. The first test treatment was performed on two nodules at a continuous wave setting of up to 10 watts. Due to a significant reduction of the nodular areas and lack of side effects, a full-surface ablative laser treatment of the nodular parts was carried out in two therapy sessions. The treatment was performed under local anesthesia using prilocainePostoperative follow-up treatment was done with dexpanthenol. The treated areas healed without visible scarring. After 8 month, there was a significant improvement of the skin condition (Fig. 1b) and the patient was very satisfied with the outcome.

### Case 2

**Fig. 2:**
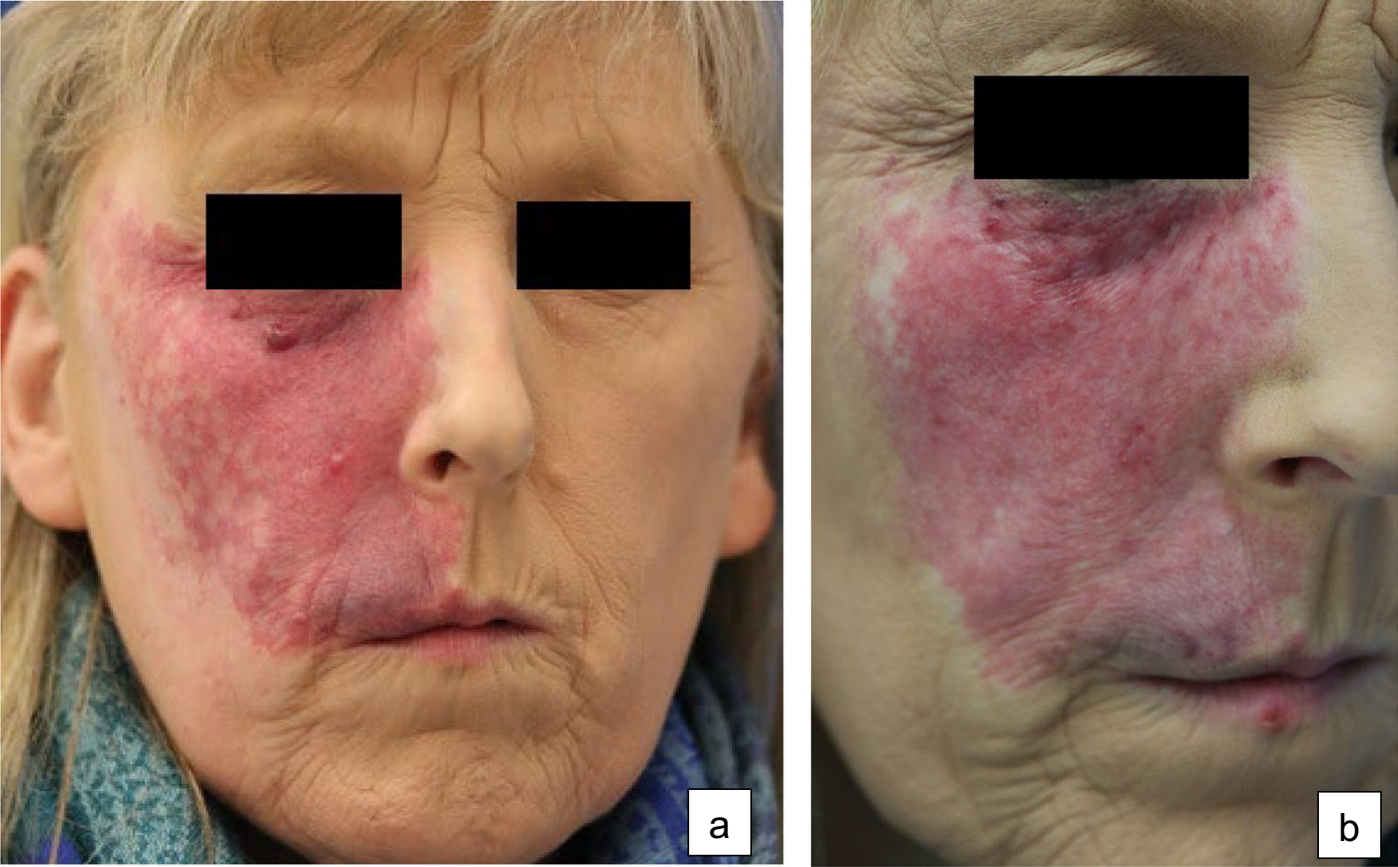
Case 2: A 64-year-old woman with congenital nevus flammeus on the right side of her face. **a** Before treatment. **b** After treatment

A 64-year-old woman presented with a PWS on the right side of her face. There were no indications for the presence of an SWS. In the past, several laser therapy sessions using a pulsed dye laser (595 nm) were carried out that led to a moderate reduction in redness. Despite the laser treatment, nodular areas developed over time. Due to the reduced quality of life, the patient underwent a total of 11 sessions using a pulsed Nd: Yag laser (1064 nm) at a setting of initially 100 J/cm² up to 160 J/cm² with a pulse duration of 90 ms and a spot size of 3 mm. Because the treatment proved ineffective (Fig. 2a) we decided to perform a treatment using CO2 laser ablation at a continuous wave setting of up to 10 watts. After four weeks, a significant flattening was observed. We then carried out a full-surface ablative laser treatment of the nodular parts in a single therapy session. The therapy was done under local anesthesia, with a follow-up with dexpanthenol-containing topicals. After laser treatment, the nodular areas of the nevus flammeus became flatter without notable side effects (Fig. 2b).

## Discussion

PWS may undergo a nodular transformation with increasing age of the patient. The percentage of patients affected ranges from about 10–44% depending on whether patients have received laser treatment or not [12]. The development of nodules is primarily related to an increasing vascular dilation and endothelial cell proliferation. Immunohistochemically, an increased expression of vascular endothelial growth factor (VEGF), matrix metalloproteinase-9 (MMP-9), angiopoietin-2 (ANG-2) and basic fibroblast growth factor (bFGF) was demonstrated in hypertrophic PWS [19].

Some guidelines recommend the use of a pulsed 1064 nm Nd: YAG laser in nodular transformation [20]. This was the initial treatment of choice in both of our patients. The 1064 nm Nd: YAG laser is a non-ablative laser with a wavelength of 1064 nm. Light from a laser of this wavelength can penetrate deeper into the skin, heating up the skin without affecting the epidermis [20]. The use of the Nd: YAG laser may cause a reduction in vascular density within the nodular components, however, the Nd: YAG laser showed no effect in the two presented cases.

In contrast, the CO_2_ laser (10,600 nm) is well suited for tissue vaporization due to its high absorption in water [21]. The resulting wound re-epithelializes starting from the remaining follicle and sweat gland parts [20]. Due to the pronounced tissue hypertrophy, we decided to treat our patients with an ablative laser. There is limited literature regarding the use of a CO_2_ laser in treating PWS [22, 23]. Using the CO2 laser, the nodular parts in our patients could be removed down to the skin level without subsequent scarring. In addition, the superficial thermal necrosis proved advantageous in terms of hemostasis as no postoperative bleeding occurred.

To clarify the risk of potential complications, it is advisable to first carry out a test treatment on one nodule before doing a full-surface treatment. Such complications after CO_2_ laser ablation include scars, impaired wound healing, post-inflammatory hyperpigmentation, hypo- or depigmentation, and persistent erythema [20]. Correct aftercare is also important, for example with topicals containing dexpanthenol, which have shown a positive effect on wound healing [20]. Consistent sun protection should also be ensured [20].

In conclusion, we regard Nd: YAG Laser as the treatment of choice for nodular PWS with high vascular component. The CO_2_ laser could be a treatment option for nodular lesions with predominantly connective tissue transformation. The CO_2_ laser may be particularly suitable for treating those nodular PWS that have undergone pre-treatments with the pulsed Nd: YAG laser without sufficient effect and therefore probably consist mainly of connective tissue.

## Data Availability

No datasets were generated or analysed during the current study.
